# Monocarboxylate transporter upregulation in induced regulatory T cells promotes resistance to anti-PD-1 therapy in hepatocellular carcinoma patients

**DOI:** 10.3389/fonc.2022.960066

**Published:** 2022-07-28

**Authors:** Jinren Zhou, Qing Shao, Yunjie Lu, Yu Li, Zibo Xu, Bo Zhou, Qiuyang Chen, Xiangyu Li, Xiaozhang Xu, Yufeng Pan, Zhenhua Deng, Yiming Wang, Yue Yu, Jian Gu

**Affiliations:** ^1^ Liver Transplantation Center, First Affiliated Hospital of Nanjing Medical University, Nanjing, China; ^2^ Department of Hepatobiliary Surgery, The First People's Hospital of Changzhou, The Third Hospital Affiliated to Soochow University, Changzhou, China; ^3^ Department of General Surgery, Ningbo Medical Center Lihuili Hospital, Ningbo University, Ningbo, China; ^4^ School of Medicine, Southeast University, Nanjing, China

**Keywords:** hepatocellular carcinoma, monocarboxylate transporter, lactate, regulatory T cells, treatment resistance

## Abstract

**Background:**

Programmed cell death-1 (PD-1) immune checkpoint inhibitors are not effective in treating all patients with hepatocellular carcinoma (HCC), and regulatory T cells (Tregs) may determine the resistance to anti-PD-1 therapy.

**Methods:**

Patients were divided into two groups based on the clinical efficacy of anti-PD-1 therapy. Flow cytometry was used to determine the phenotype of CD4+, CD8+, and Tregs in peripheral blood mononuclear cells (PBMCs). CD4+CD45RA+T cells were sorted to analyze Treg differentiation and function.

**Results:**

No significant differences were found between resistant and sensitive patients in the percentage of CD4+ T cells and Tregs in PBMCs or the differentiation and function of induced Tregs (iTregs). However, iTregs from resistant patients presented higher monocarboxylate transporter (MCT) expression. Lactate induced more iTregs and improved OXPHOS levels in the resistant group. MCT1 and MCT2 were highly expressed in tumor-infiltrating Tregs, and patients with higher MCT1 expression had worse clinical outcomes. Combinatorial therapy with MCT antibody and anti-PD-1 therapy effectively inhibited tumor growth.

**Conclusion:**

MCT and its downstream lactate signal in Tregs can confer anti-PD-1 resistance and may be a marker of poor prognosis in HCC.

## Introduction

Hepatocellular carcinoma (HCC) is a common malignancy worldwide (1). Except for surgical resection and liver transplantation, effective anti-tumor drugs are limited, and the prognosis of patients with HCC is relatively poor. Immunotherapy alone or combined with targeted therapy of HCC has received considerable attention in clinical research of HCC (2). However, a significant number of patients remain resistant to immunotherapy. Studying the immune microenvironment in these patients could provide a theoretical basis for the underlying mechanism of resistance and identify potential targets for clinical treatment (3).

Regulatory T cells (Tregs) play an important role in maintaining immunological homeostasis (4). Tregs are divided into two main subsets: thymus-derived natural Tregs (nTregs) and induced Tregs (iTregs). Tregs are present in the tumor microenvironment (TME) and can crosstalk with anti-tumor cells, such as infiltrating lymphocytes, macrophages, and natural killer cells (5). They utilize multiple mechanisms to achieve immunosuppression, including the inhibition of antigen-presenting cell (APC) maturation, inhibitory cytokines secretion, and cytotoxic granzyme and perforin production. Although tTregs can be recruited into tumors to suppress the effective T cells, most tumor-infiltrating Tregs are iTregs, which can be induced by interleukin (IL)-2 and transforming growth factor beta (TGF-β) and express forkhead box P3 (Foxp3) and cytotoxic T-lymphocyte-associated protein 4 (CTLA-4) (6). Tregs suppress anti-tumor immune responses, and their infiltration into tumors affects prognosis. The removal of Treg cells can enhance anti-tumor immunity, but their depletion may also trigger detrimental autoimmune responses (7). The response of Tregs to immune checkpoint inhibition (ICI) differs from that induced by other immune cells (8). A recent study showed that tumor resistance to anti-programmed cell death-1 (anti-PD-1) therapy might be related to an increase in CTLA-4^+^Tregs (9). Moreover, the targeting of CCR8 to deplete tumor-infiltrating Tregs may elicit antitumor immunity and synergize with anti-PD-1 therapy (10).

The proton-coupled monocarboxylic acid transporter (MCT) catalyzes the transmembrane movement of essential monocarboxylate salts, such as L-lactic acid, ketone bodies, and pyruvate, which are essential in many cellular processes. MCT also catalyzes lactate exchange between tumors and the circulation (11) and has become a target for cancer treatment because of its enhanced expression in various tumors (12). Here, we demonstrated that higher expression of MCT and sensitivity to lactate may upregulate the function of Tregs in the TME, which may explain the poor prognosis of patients with HCC who are resistant to anti-PD-1 therapy. We propose that by evaluating MCT expression and suppressing the MCT signaling, the efficacy of therapeutic strategies can be improved.

## Materials and methods

### Clinical study and the patients

We selected 10 patients with a clear clinical diagnosis of HCC between January 2020 and December 2021 as research participants. Some patients experienced tumor recurrence and underwent liver resection within <1 year. Patients received four treatments of anti-PD-1 therapy every 3 weeks (camrelizumab, Jiangsu Hengrui Medicine) and 9 weeks of apatinib. Patients with decreased tumor volume were included in the sensitive group; patients with a 25% increase in tumor volume were included in the resistant group. Kaplan–Meier survival curves were used to estimate overall survival (OS) and disease-free survival (DFS). Survival curves were exported using GraphPad Prism software (GraphPad Software, San Diego, CA, USA). The local ethics committee of the First Affiliated Hospital of Nanjing Medical University (Jiangsu Province Hospital) approved this study (2020-SRFA-377).

### Specimen collection

Fasting blood samples were collected at 7 a.m. before treatment. Blood samples (10–20 ml) were used to monitor T-cell function and phenotype in peripheral blood mononuclear cells (PBMCs). PBMCs were prepared from heparinized venous blood by Ficoll–Hypaque density gradient centrifugation. To evaluate the proportion of CD8^+^ T cells and CD4^+^ T cells, PBMCs were surface stained with CD8 and CD4. PBMCs were stained with CD4, CD25, and CD127, and CD4^+^CD25^+^CD127^−^ cells were considered to be Tregs. Naive human T cells were extracted from PBMCs by gating CD4^+^CD45RA^+^ cells (>97% purity). iTregs were activated, induced, and expanded with TGF-β (1ng/ml), IL-2 (100 U/ml), and anti-CD3/CD28 beads (one bead per cell) for 7 days.

Tregs were sorted for the next analysis after tissue lymphocytes were extracted (P5700 kit, Solarbio). In HCC and para-cancerous tissues, we analyzed MCT and lactate expression levels using Western blot (Abcam, Cambridge, UK) and a lactic acid content assay kit (BL868A, Biosharp), respectively.

### Animal models and tumor generation

Hepa1-6 tumor cells (5 × 10^6^) were injected into the armpits of C57L/B6 and Rag1 −/− 6–8-week-old mice (purchased from the Model Animal Research Center of Nanjing University, Nanjing, China). After 5 days, 10 nM anti-MCT1/2 (AR-C155858, MCE) and 200 μg anti-PD1 (HY-P9971, MCE) were injected into the axilla of the experimental group every 2 days, while the control group was injected with saline. Tumor volume was measured using a digital caliper. After four injections, all mice were euthanized, and the tumor tissues were removed.

### Flow cytometry

Human-specific monoclonal antibodies, including CD4 (A161A1), CD25 (BC96), Foxp3 (206D), and CD45RA (HI100), were purchased from BioLegend (UK), and CD127 (A019D5) was purchased from BD Pharmingen (USA). Foxp3 (206D) was used for intracellular flow cytometry assays, while the others were used for surface staining. A MACSQuant Analyzer 10 (Miltenyi Biotec, Germany) was used for detection, and results were analyzed using FlowJo software (FlowJo, Ashland, OR, USA).

### Western blot analysis

Proteins were collected from harvested cells, and a Pierce bicinchoninic acid assay (Thermo Fisher Scientific, UK) was used to determine their concentrations. The proteins were resolved and transferred onto polyvinylidene fluoride membranes. The following antibodies were used: MCT1, MCT2, LDH, anti-GAPDH, anti-P85 (loading control), and tubulin (Abcam, Cambridge, UK). Data were displayed using the Kodak autoradiography film (Kodak XAR film, USA).

### Immunofluorescence staining

MCT1, MCT2, and Foxp3 were identified by immunofluorescence using rabbit anti-human MCT1, anti-human MCT2, and anti-human Foxp3 mAbs (Cell Signaling Technology, Danvers, MA, USA). Images were obtained using an inverted microscope (Olympus, Tokyo, Japan) and analyzed using ImageJ software (National Institutes of Health, Bethesda, MD, USA).

### Cell metabolism measurement

An XF96 analyzer (Agilent Technologies, Santa Clara, CA, USA) was used to measure Treg cell metabolism. First, Tregs were inoculated onto CellTak 96-well plates at 2×10^5^ cells/well and centrifuged. Different reagents were added to measure the oxygen consumption rate (OCR), including 5 mM oligomycin, 1.5 μM FCCP, 1 μM rotenone, and 1 μM antimycin A.

### ELISA

We measured the levels of IL-10, TGF-β, and other cytokines using ELISA kits (BioLegend, UK) according to the manufacturer’s instructions.

### Statistical analysis

Data were analyzed and visualized using GraphPad Prism 6.0 (GraphPad Software). Count data were analyzed using a paired t-test and linear and nonlinear regression analysis. A log-rank test was used to analyze differences in the Kaplan–Meier survival curves. Statistical significance was set at p <0.05.

## Results

### Role of Treg phenotype and cytokine expression in the peripheral blood of anti-PD-1-resistant patients

**Figure 1:**
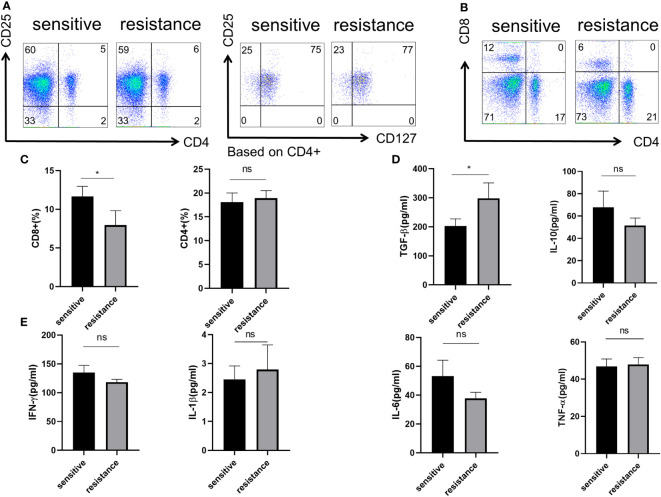
Role of Treg phenotype and cytokine expression in the peripheral blood of anti-PD-1-resistant patients. **(A)** Representative flow plot for CD4, CD25, and CD127 expression in the periphery blood of sensitive and resistant patients. **(B)** Representative flow plot for CD4 and CD8 expression in the periphery blood of sensitive and resistant patients. **(C)** The proportion of CD4+ and CD8+ T cells between both groups. The expression of anti-inflammatory cytokines **(D)** and inflammatory cytokines **(E)** from the serum was evaluated through ELISA. The bar shows the mean ± SEM of the levels of indicated proteins (sensitive patients n=4; resistant patients n=4). *p < 0.05. ns, non-significant.

As the efficiency of the targeted and anti-PD-1 therapy is highly dependent on the immune status of the liver, we first determined the phenotype of Tregs, CD4+, and CD8+ T cells in the blood. Flow cytometry showed no difference in the percentages of CD4^+^ T cells and CD4^+^CD25^+^CD127^−^ Tregs (**Figure 1A**). However, more CD8^+^ T cells were present in the sensitive group, indicating that the efficacy of co-treatment might be partially determined by CD8^+^ T cells (**Figures 1B**, **C**). We evaluated cytokine expression in the blood serum and found that, for anti-inflammatory cytokines, TGF-β was upregulated in the resistant group, and IL-10 levels were similar between the sensitive and resistant groups (**Figure 1D**). The expression of inflammatory cytokines, such as TNF-α, interferon-gamma (IFN-γ), IL-1, and IL-6, which act as anti-tumor effectors, did not vary between the sensitive and resistant groups (**Figure 1E**). Tregs can differentiate into nTregs and iTregs (13) and suppress CD8^+^ T cells (14, 15). As no differences in nTregs were observed under resting conditions between the sensitive and resistant groups, naive T cells were examined for their ability to differentiate into iTregs.

### Naive T cells regulated iTreg induction and function *in vitro*

**Figure 2:**
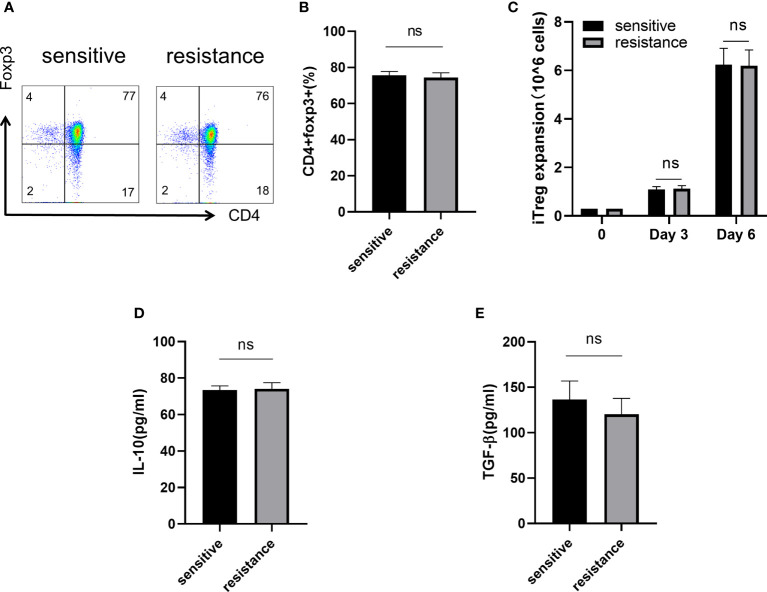
Naive T cells regulated iTreg induction and function *in vitro.* Representative flow plot **(A)** and the frequency **(B)** of Foxp3 expression in iTregs of sensitive and resistant patients. The expansion ability of iTregs. **(C)** The expression of anti-inflammatory cytokines including IL-10 **(D)** and TGF-β **(E)** from the serum was evaluated through ELISA. The bar shows the mean ± SEM of the levels of indicated proteins (sensitive patients n=4; resistant patients n=4). ns, non-significant.

CD4^+^CD45RA^+^ naive T cells were sorted and differentiated into iTregs (*Section 2.2*). After 6 days, flow cytometry showed similar level of expression for Foxp3 in both groups (**Figures 2A**, **B**). We counted the absolute number of Tregs after 3 and 6 days to assess iTregs activity in the sensitive and resistant groups, but no differences were observed between the groups (**Figure 2C**). Tregs maintain an immunosuppressive environment through the expression of specific cytokines, which is important for the establishment of a tumor-dominant microenvironment. We monitored the expression of IL-10 and TGF-β using ELISA and found no discrepancy in the secretory ability of Tregs between the sensitive and resistant groups (**Figures 2D**, **E**).

### Induction of iTregs by lactate in the resistance group

**Figure 3:**
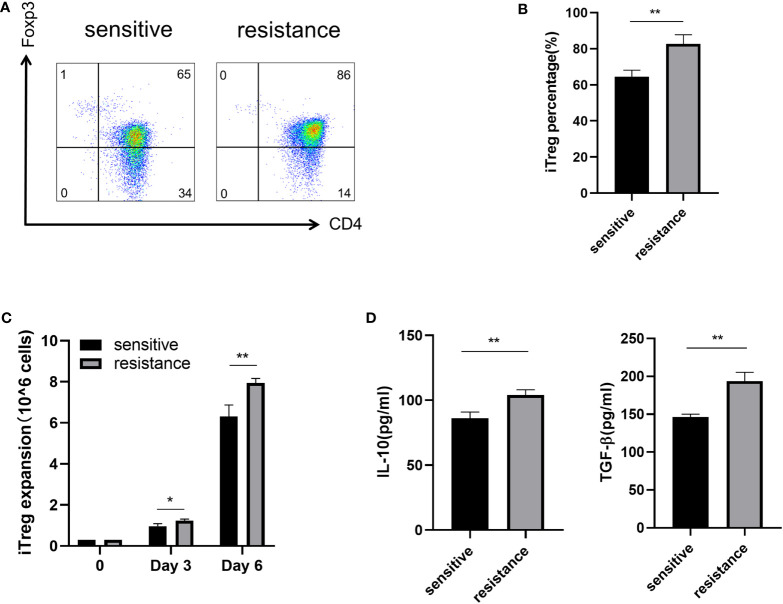
Induction of iTregs by lactate in the resistance group. Representative flow plot **(A)** for and the frequency **(B)** of Foxp3 expression in iTregs with the addition of lactate (10 mM) from sensitive and resistant patients. **(C)** The expansion ability of iTregs. **(D)** The expression of anti-inflammatory cytokines including IL-10 and TGF-β from the serum was evaluated through ELISA. The bar shows the mean ± SEM of the levels of indicated proteins. (sensitive patients n=4; resistant patients n=4). *p < 0.05; **p < 0.01.

Lactate improves the function of iTregs in tumors and is one of the most important mechanisms mediating immunosuppression and tumor immune escape (16, 17). We treated the sensitive and resistant groups with lactate (10 mM) to investigate its effects on iTreg induction. Lactate improved the expression of Foxp3 in the resistant group but not in the sensitive group (**Figures 3A**, **B**). In addition, we observed that lactate improved the proliferation of iTregs in the resistant group compared with that in the sensitive group (**Figure 3C**). As expected, the expression of IL-10 and TGF-β was also upregulated, which corresponded with the above findings (**Figure 3D**).

### Foxp3 was upregulated in the iTregs of the resistant group in an MCT-dependent manner

**Figure 4:**
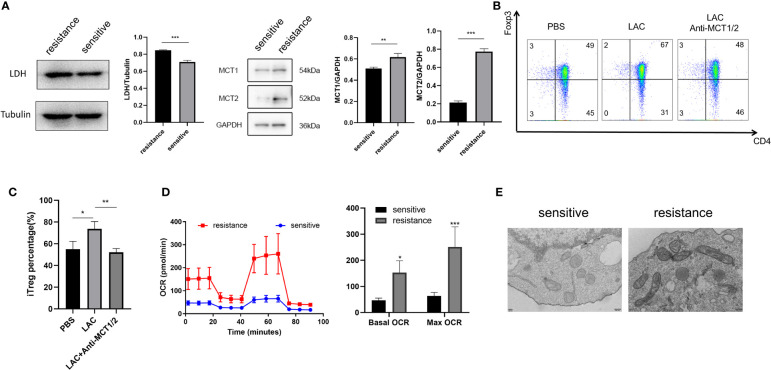
Foxp3 was upregulated in the iTregs of the resistant group in an MCT-dependent manner. **(A)** Representative blot plot for expression of LDH, MCT1, and MCT2 in both groups. Representative flow plot **(B)** for and the frequency **(C)** of Foxp3 expression in iTregs with the addition of lactate (10 mM) and inhibitors of MCT1 and MCT2 in resistance patients. **(D)** Oxygen consumption per unit time of two groups. **(E)** Representative plot for mitochondrial morphology observed through electron microscopy. The bar shows the mean ± SEM of the levels of indicated proteins. *p < 0.05; **p < 0.01; ***P<0.001.

Lactate acidifies the extracellular space and maintains intracellular pH homeostasis inside the cell through MCT (18). To investigate why iTregs could be induced more effectively by lactate treatment in the resistant group, we examined the expression of MCT1 and MCT2, which are the most important contributors to the regulation of tumor intracellular pH and induction of extracellular acidosis (19). Western blot analysis revealed higher MCT1 and MCT2 expression levels in the resistant group (**Figure 4A**). Although lactate improved Foxp3 expression, the addition of MCT1 and MCT2 antibodies eliminated this effect and reduced the expression of Foxp3 (**Figures 4B**, **C**). Because lactate alters the metabolism of Tregs (16), we evaluated the bioenergetics changes after lactate treatment and found that Tregs in the resistance group exhibited an increased basal OCR and maximal respiratory capacity (evident after exposure to FCCP), indicating increased mitochondrial activity (**Figure 4D**). Electron microscopy also revealed that mitochondria were fused (**Figure 4E**) and had greater metabolic capacity in the resistant group (20).

### Higher MCT1 and MCT2 expression was observed in tumor-infiltrating Tregs in resistant patients

**Figure 5:**
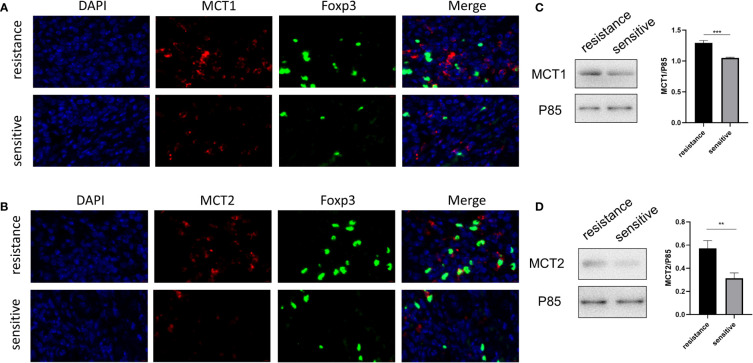
Higher MCT1 and MCT2 expression was observed in tumor-infiltrating Tregs in resistant patients. Representative plot for expression of MCT1 and MCT2 in Tregs in resected tumor samples from both groups under the confocal microscopy **(A, B)** and Western blot **(C, D)**. **p < 0.01; ***P<0.001.

The *in vitro* expression of LDH, MCT1, and MCT2 was upregulated in the iTregs of the resistant group but not the sensitive group. In resected tumor samples, we observed higher levels of MCT^+^ cells by immunofluorescence staining (**Figures 5A**, **B**) and Western blot analysis (**Figures 5C**, **D**) in resistant than sensitive patients.

### MCT1 and MCT2 was a poor prognosis marker of aggressive HCC

**Figure 6:**
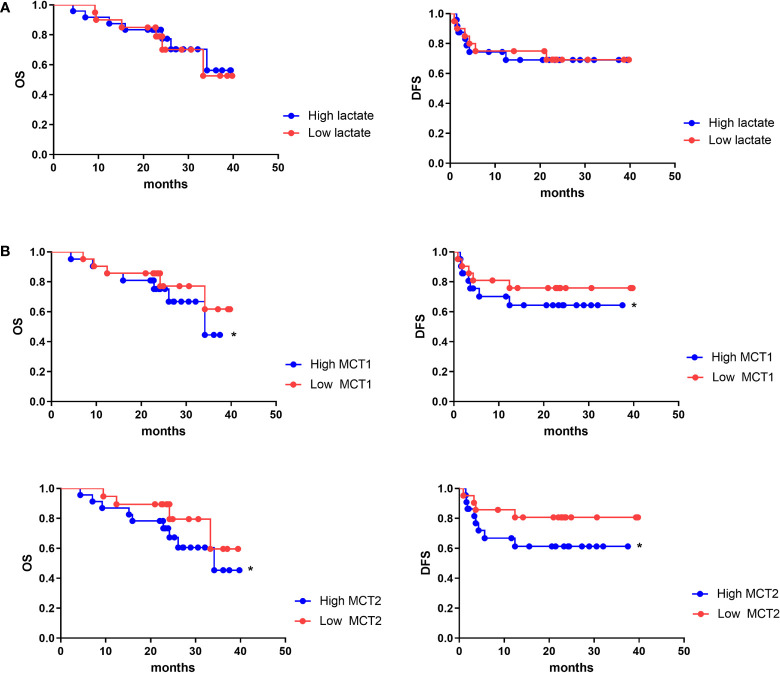
MCT1 and MCT2 was a poor prognosis marker of aggressive HCC. Kaplan–Meier survival analyses were conducted to assess the influence of lactate **(A)** and MCT1 and MCT2 **(B)** on overall survival and disease-free survival of HCC. *p < 0.05.

As lactate functions in cells in the presence of MCT, we further evaluated the role of lactate or MCT in the prognosis of HCC. We enrolled 43 patients who underwent HCC resection surgery at our center in 2017, who were then divide into high- and low-lactate groups according to Treg lactate content. Although lower-lactate patients had slightly lower OS rates than those with high-lactate patients, no statistically significant differences were observed in the OS and DFS between the groups (**Figure 6A**). Patients were also grouped according to their MCT1 and MCT2 expression levels in Tregs (**Table 1**). As expected, patients with higher MCT1 and MCT2 levels had poorer prognosis in terms of OS and DFS rates than those with lower levels (**Figure 6B**).

**Table 1:** Patient characteristics.

	MCT1 high(n=20)	MCT1 low (n=21)
Gender(male/female, n)	17/3	17/4
Age(years, mean ± SD)	59.30 ± 10.70	57.48 ± 8.87
AFP(ng/ml, mean ± SD)	334.55 ± 453.50	223.00 ± 417.60
tumor size(cm, mean ± SD)	5.31 ± 2.44	6.00 ± 2.42
MVI [n(%)]	10(50.0)	11(52.4)
Liver function(Child score)	5.10 ± 0.30	5.19 ± 0.50

AFP, alpha-fetoprotein; MVI, microvascular invasion.

### Co-treatment of MCT and PD-1 inhibitors reduced tumor growth by downregulating Tregs and upregulating anti-tumor cytokine expression

**Figure 7:**
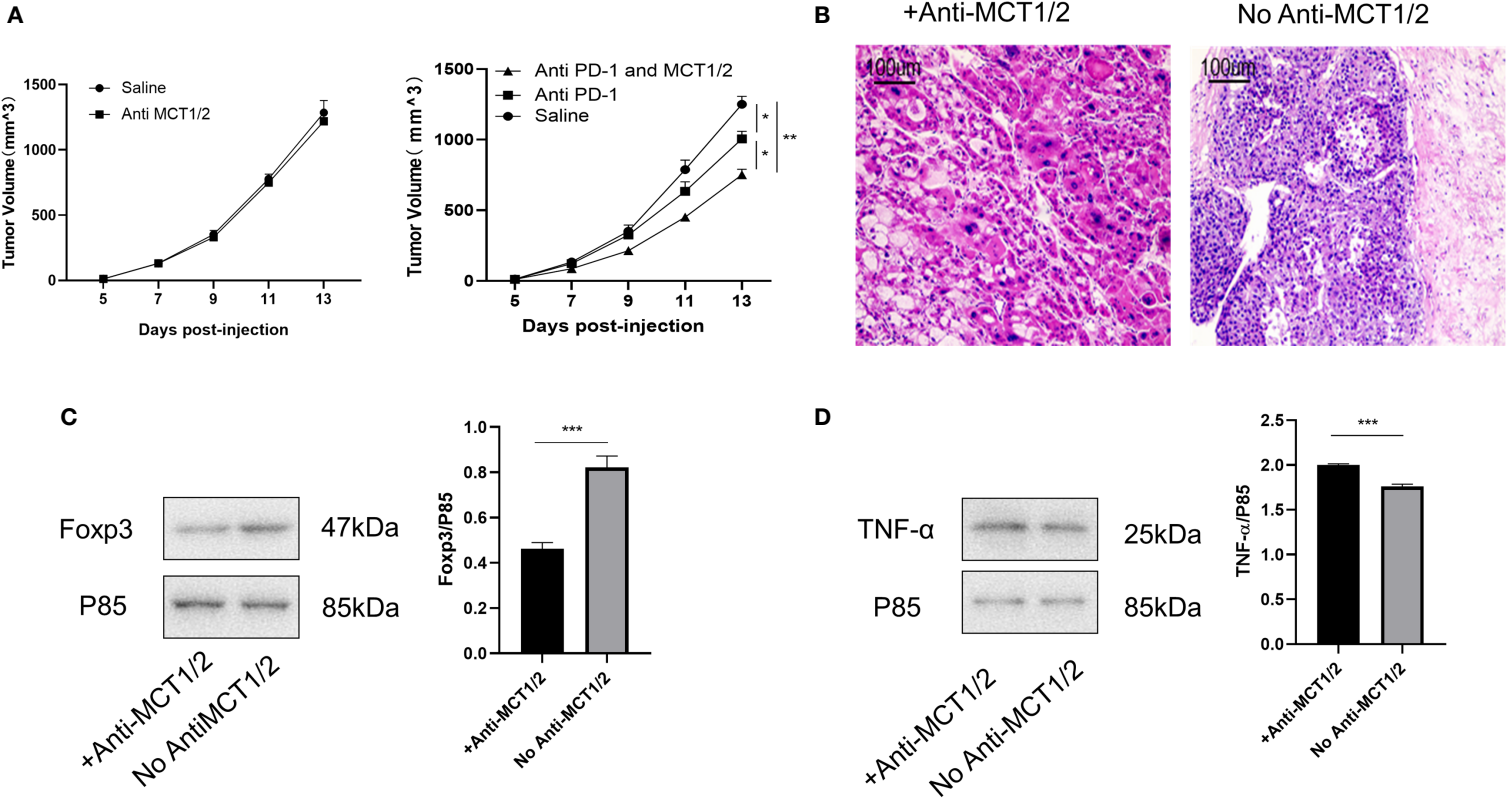
Co-treatment of MCT and PD-1 inhibitors reduced tumor growth by downregulating Tregs and upregulating anti-tumor cytokine expression. **(A)** Tumor growth in different groups post-injection (left Rag1^−/−^ mice and right C57L/B6 mice). **(B)** HE staining of tumor from different groups. **(C, D)** Western blot for expression of Foxp3 and TNF-α in different groups. The result is representative of three independent experiments. Data were mean ± SD of three independent experiments (each group, n=3). *p < 0.05; **p < 0.01; ***P<0.001.

Lastly, we tested whether co-treatment with MCT inhibitor would promote the curative effects of anti-PD-1 therapy. The tumor volume in mice receiving the co-treatment was significantly smaller than that in mice injected with anti-PD-1 alone (**Figure 7A**). Hematoxylin and eosin staining revealed the presence of more tumor-infiltrating lymphocytes in mice injected with the MCT inhibitor. Moreover, the proportion of cells undergoing nuclear fragmentation and cell death increased. This indicated that the anti-tumor inflammatory response was much more intensive, thus inhibiting the growth of the tumor (**Figure 7B**). Western blot analysis confirmed that Foxp3 and TNF-α expression was markedly decreased and increased, respectively, in the co-treated group (**Figures 7C**, **D**).

## Discussion

HCC is a lethal malignancy that originates from hepatocytes differentiation (21). In China, 466,000 new cases of HCC and 422,000 related deaths occur annually (22). In addition, only 10%–15% of patients meet the criteria for surgical excision due to disease severity (23). The current treatments for primary HCC are diverse and include radical surgical resection, multi-target antitumor drugs, combined chemotherapy, trans-arterial chemoembolization, radiofrequency ablation, and anhydrous alcohol injection (24). Due to heterogeneity between HCC cases and the complexity of immunoregulatory mechanisms in the TME, targeted combined immunotherapy is currently the most efficient clinical cancer treatment, other than surgery.

Among the possible targets for immunotherapies, PD-1/PD-L1—a major immune checkpoint (9, 16)—is widely used in clinical treatments in China. However, anti-PD-1 therapy is sometimes ineffective (25). Single-agent anti-PD-1 therapy using nivolumab achieved an objective response rate (ORR) of 20% (95% confidence interval [CI], 15%–26%) and a disease control rate (DCR) of 64% (95% CI, 58%–71%) (26). The CheckMate-459 trial, a multi-center phase III randomized trial, found that nivolumab was not a suitable alternative to sorafenib (27). Even with targeted combined immunotherapy, ORR and DCR were <50% (28).

In this study, we assessed the underlying mechanisms of resistance to anti-PD-1 therapy according to the generation, activation, and function of iTregs. Our cytometry data did not show a difference in the immune cell phenotypes of PBMCs. The Treg induction rate and suppressive functions were also similar in both sensitive and resistant groups *in vitro*. We hypothesized that the TME determines the different functions of Tregs and the prognosis of anti-PD-1 therapy. Concurrently, in the no-TME condition, Tregs did not show an obvious difference in function, which may be caused by differences in between the *in vitro* and *in vivo* environments.

A recent study showed that lactate, a metabolite produced by tumors, is critical for regulating the tumor environment, especially the expression of Tregs and macrophages (29). Lactate can regulate the number, induction, inhibitory function, and metabolic mechanisms of Tregs in the TME (17). Tregs acquire higher PD-1 expression than effector T cells in the TME, which is mediated by MCT1. PD-1 blockade activates PD-1-expressing Tregs, which leads to treatment failure (30). Therefore, we investigated whether iTreg induction varied between the two groups. Lactate induced more iTregs in the resistant than in the sensitive group. As MCT can promote lactate transport both inside and outside Tregs, we also evaluated MCT expression in iTregs and resected tumors, which showed that resistant patients had higher MCT expression than sensitive patients. High MCT expression and sensitivity to lactate can upregulate the function of Tregs in the TME. The blocking of MCT abolished Foxp3 upregulation by lactate in the resistant group. Finally, we demonstrated that co-treatment with an MCT neutralizer and anti-PD-1 was effective in murine subcutaneous tumor models. As MCT expression is important for Treg generation in the TME, further studies should be performed to analyze the underlying mechanism of T cells with both high and low expression.

In conclusion, we have shown that MCT and its downstream lactate signals in Tregs may result in resistance to anti-PD-1 therapy. Therefore, the expression of MCT in the iTregs from PBMCs or resected tumors can be used to predict prognosis before anti-PD-1 therapy. We acknowledged that our findings are based on a relatively small sample size; thus, future studies should consider larger multi-center cohorts. Our study provides insights into the modulation of immunosuppression in HCC and novel targets for the development of intuitive therapeutic strategies.

## Data availability statement

The original contributions presented in the study are included in the article/supplementary material. Further inquiries can be directed to the corresponding authors.

## Ethics statement

The animal study was reviewed and approved by the Institutional Animal Care and Research Advisory Committee of Nanjing Medical University. Written informed consent was obtained from the individual(s) for the publication of any potentially identifiable images or data included in this article.

## Author contributions

JZ, YJ L, YY, and JG designed the experiments. JG and JZ provided the study materials or patients. QS, YL, BZ, ZX, XL, and XX performed the experiments and interpreted the data. QC, YP, ZD, and YW revised the manuscript. All authors read and approved the final manuscript.

## Funding

This work was supported by the National Natural Science Foundation of China (81971504), Natural Science Foundation of Jiangsu (BK20201486), Post-Doctoral Special Foundation of China (2020M670065ZX), Post-Doctoral Foundation of Jiangsu Province (2020Z021), Medical and Health Science and Technology Program of Zhejiang Province (2021KY1036), Changzhou Society Development Funding (CE20205038), and the lifting Project of 2021 Young Scientific and Technological Talents in Changzhou.

## Conflict of interest

The authors declare that the research was conducted in the absence of any commercial or financial relationships that could be construed as a potential conflict of interest.

## Publisher’s note

All claims expressed in this article are solely those of the authors and do not necessarily represent those of their affiliated organizations, or those of the publisher, the editors and the reviewers. Any product that may be evaluated in this article, or claim that may be made by its manufacturer, is not guaranteed or endorsed by the publisher.
